# Supplementary material for: Lipid hydrogen isotope compositions primarily reflect growth water in the model archaeon Sulfolobus acidocaldarius
Source: Appl Environ Microbiol. 2025 Mar 25;91(4):e01983-24. doi: 10.1128/aem.01983-24 (PMC12016520; doi:10.1128/aem.01983-24)
Supplement: Table S1 — Summary of culture conditions and descriptive growth statistics for S. acidocaldarius grown in media prepared with 2H-labeled waters. RPM refers to shaking rate. [file aem.01983-24-s0002.docx]

#### **Table S1.** Summary of culture conditions and descriptive growth statistics for S. acidocaldarius grown in media prepared with ^2^H-labeled waters. RPM is shaking rate.

|  |  |  | **Growth Conditions** | | | | **Growth Rate (hour^-1^)** | | **Doubling Time (Hours)** | | **Max OD** | |
| --- | --- | --- | --- | --- | --- | --- | --- | --- | --- | --- | --- | --- |
| **Experiment** | **δ^2^H_W_ (‰)** | **N** | **T °C** | **pH** | **RPM*** | **O_2_ %** | **Mean** | **sd** | **Mean** | **sd** | **Mean** | **sd** |
| Water Label | -362 | 3 | 70 | 3 | 200 | air | 0.10 | 0.00 | 6.68 | 0.10 | 0.72 | 0.01 |
|  | -48 | 3 | 70 | 3 | 200 | air | 0.08 | 0.00 | 8.64 | 0.27 | 0.68 | 0.01 |
|  | +419 | 3 | 70 | 3 | 200 | air | 0.08 | 0.00 | 8.40 | 0.10 | 0.65 | 0.01 |
