# Supplementary material for: Lipid hydrogen isotope compositions primarily reflect growth water in the model archaeon Sulfolobus acidocaldarius
Source: Appl Environ Microbiol. 2025 Mar 25;91(4):e01983-24. doi: 10.1128/aem.01983-24 (PMC12016520; doi:10.1128/aem.01983-24)
Supplement: Table S2 — Descriptive statistics for individual biphytanyl (BP) lipids derived from iGDGTs for S. acidocaldarius grown in media prepared with 2H-labeled waters. [file aem.01983-24-s0003.docx]

**Table S2***.* Descriptive statistics for individual biphytanyl (BP) lipids derived from iGDGTs for *S. acidocaldarius* grown in media prepared with ^2^H-labeled waters. Mean and sd include biological replication (N ≥ 1) and technical replication (e.g., multiple injections, n ≥ 3). BP-3 was present in too low abundances to get reliable isotope data. Abundance-weighted means are shown for δ^2^H_BP_ and ^2^ε_L/W_ values with propagated error. RI-BP is the BP Ring Index.

|  |  |  | **BP-0** | | | | | | **BP-1** | | | | | | **BP-2** | | | | | |
| --- | --- | --- | --- | --- | --- | --- | --- | --- | --- | --- | --- | --- | --- | --- | --- | --- | --- | --- | --- | --- |
| **Experiment** | **δ^2^H_W_ (‰)** | **N** | **Rel Abund** | **sd** | **δ^2^H_BP_ (‰)** | **sd** | **²ε_L/W_**  **(‰)** | **sd** | **Rel Abund** | **sd** | **δ^2^H_BP_ (‰)** | **sd** | **²ε_L/W_**  **(‰)** | **sd** | **Rel Abund** | **sd** | **δ^2^H_BP_ (‰)** | **sd** | **²ε_L/W_**  **(‰)** | **sd** |
| Water Label | -362 | 2 | 0.2 | 0.0 | -469.1 | 2.7 | -165.8 | 1.6 | 0.3 | 0.0 | -467.0 | 0.6 | -162.4 | 1.8 | 0.4 | 0.0 | -472.3 | 3.2 | -170.8 | 2.4 |
|  | -48 | 1 | 0.2 | 0.0 | -288.6 | 5.5 | -253.5 | 5.8 | 0.3 | 0.0 | -296.9 | 18.6 | -261.2 | 19.6 | 0.5 | 0.0 | -298.8 | 13.1 | -263.2 | 13.7 |
|  | +419 | 2 | 0.2 | 0.0 | -65.3 | 21.1 | -342.1 | 14.3 | 0.3 | 0.0 | -27.5 | 7.6 | -315.5 | 4.7 | 0.5 | 0.0 | -15.5 | 6.0 | -307.1 | 4.8 |

(Table S2, Part 1) *continues below*

| **BP-3** | | **Abundance weighted mean** | | | | **BP-RI** | |
| --- | --- | --- | --- | --- | --- | --- | --- |
| **Rel Abund** | **sd** | **δ^2^H_BP_ (‰)** | **sd** | **²ε_L/W_ (‰)** | **sd** | **mean** | **sd** |
| 0.1 | 0.0 | -469.7 | 9.1 | -166.7 | 9.1 | 1.3 | 0.0 |
| 0.04 | 0.0 | -283.0 | 4.1 | -247.6 | 4.1 | 1.4 | 0.0 |
| 0.05 | 0.0 | -26.7 | 59.8 | -315.0 | 59.8 | 1.5 | 0.0 |

(Table S2, Part 2) */end*
